# Talar Neck Fracture after United Tibiotalar Fusion

**DOI:** 10.1155/2015/927259

**Published:** 2015-09-28

**Authors:** W. Platt, M. Welck, B. Rudge

**Affiliations:** ^1^Tunbridge Wells Hospital, Tonbridge Road, Pembury, Kent TN2 4QJ, UK; ^2^West Hertfordshire Hospitals NHS Trust, Vicarage Road, Watford WD18 0HB, UK

## Abstract

Tibiotalar arthrodesis is a well-established treatment for tibiotalar arthritis, for example, in younger high demand patients. Talar neck fractures are less common though well-recognised sequelae of foot ankle trauma. Here we present the clinical case of a 69-year-old male who presented to our institution with a nonunion of a talar neck fracture, having undergone a left tibiotalar fusion 24 years previously. To the authors' knowledge, this injury has only been described once previously in the literature. However, the original case described a fracture sustained in the very early postoperative period following tibiotalar fusion, postulated to be secondary to postimmobilisation osteopaenia or stress risers from metalwork. The aetiology in this case is likely due to axial compression transmitted to the talar neck via the calcaneus. The predisposing factors for nonunion are discussed, highlighting the importance of vigilance for this injury in any patient with concomitant tibiotalar fusion and foot trauma. The management of this patient is discussed.

## 1. Case History

A 69-year-old male presented to our institution in June 2012, suffering from left foot pain and increased limp. He had a history of bilateral successful tibiotalar fusions for ankle arthritis (left 1987; right 1996). His past medical history included tablet controlled type 2 diabetes and ischaemic heart disease. His haemoglobin A1C was 56 mmol (range 19–43). He was a nonsmoker.

Seven months prior to presentation (November 2011), he fell off a ladder from a height of six feet and landed on his left hind foot. He presented at that time to a local Emergency Department where X-rays at that time were reported as normal. He was subsequently reviewed in fracture clinic at that hospital before being discharged.

Clinical examination on presentation to our institution revealed tenderness over the dorsum of the ankle, reduced subtalar movement, and a small degree of apparent dorsiflexion and plantar flexion of the ankle. Sensation was preserved with no evidence of diabetic neuropathy. Foot pulses were palpable, although weak.

**Figure 1:**
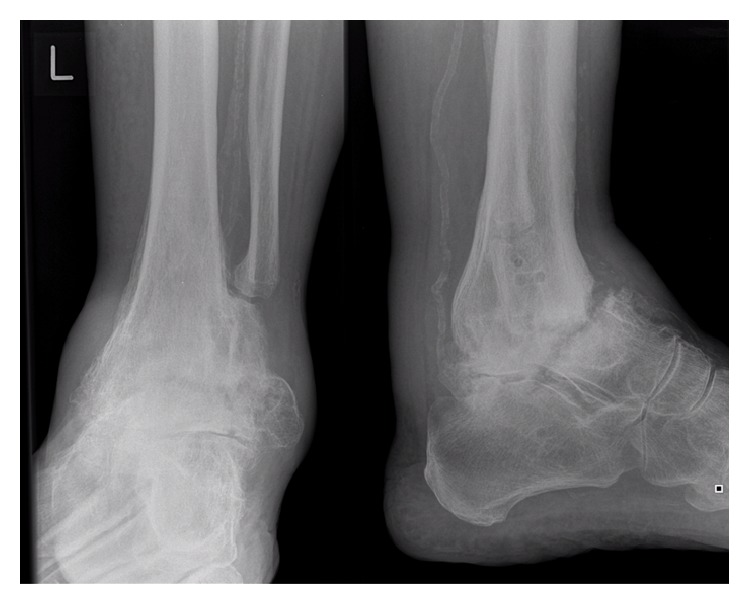
Radiograph at initial presentation to our institution, demonstrating nonunion of talar neck fracture below tibiotalar arthrodesis.

**Figure 2:**
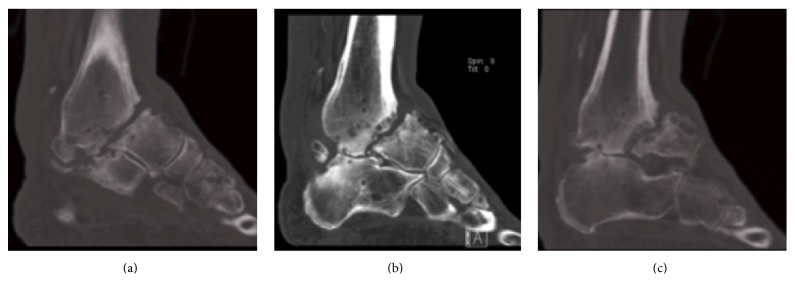
CT scan at presentation: sagittal slices demonstrate size of distal fragment. (a) Medial slice, (b) central slice, and (c) lateral slice.

**Figure 3:**
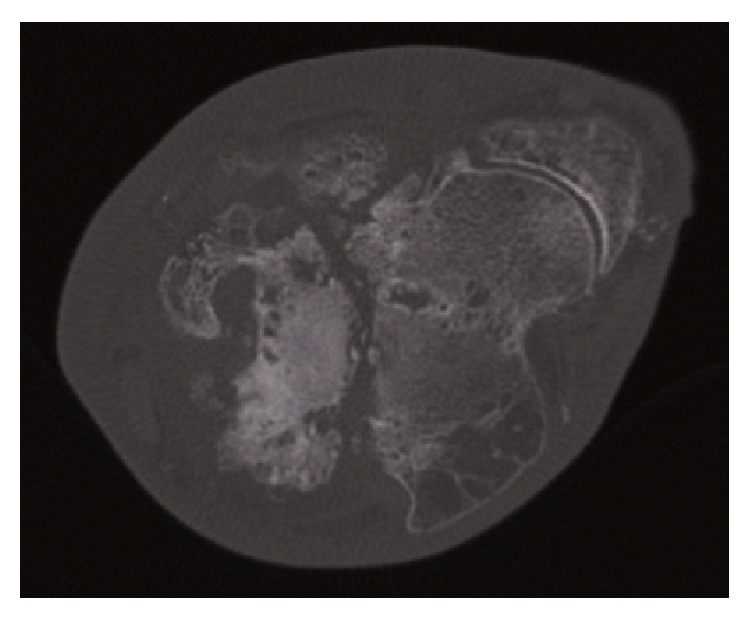
CT scan at presentation: axial slices demonstrate size of distal fragment.

Radiographs (Figure 1) revealed an established nonunion of the talar neck along with subtalar arthritis and calcification of the vessels. A computed tomography (CT) scan demonstrated an ununited, undisplaced talar neck fracture with a large fragment of talus distal to the fracture. It also confirmed a solid tibiotalar fusion and subtalar arthritis as per the plain radiology (Figures 2 and 3). The apparent ankle movement noticed on examination was due to the nonunion functioning as a pseudarthrosis.

Operative options were discussed with the patient, though prior to undertaking and surgery the patient underwent a diagnostic subtalar injection to assess the need for a simultaneous subtalar fusion. The patient's circulatory status was reviewed in vascular clinic due to arterial calcification seen on imaging and a chronic cellulitic appearance to his legs. Advice received from the vascular surgeons was to cover the patient with low dose prophylactic penicillin. His fitness for anaesthesia was optimised including cardiology workup and the patient came to operation in July 2013. He underwent an open reduction and internal fixation of talar neck nonunion and simultaneous subtalar fusion of the left ankle.

**Figure 4:**
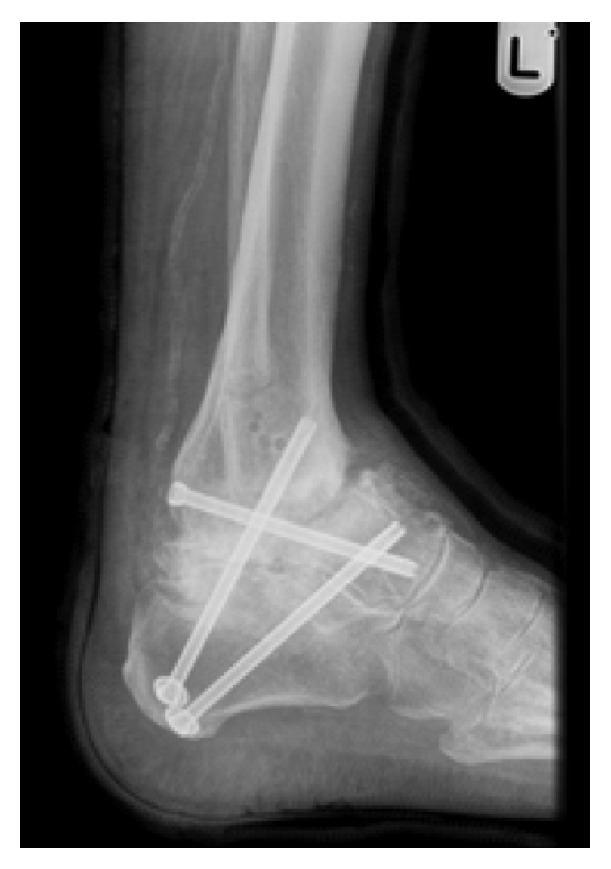
Immediate postoperative radiograph.

The operation was performed through a single incision lateral approach. The subtalar joint and talar neck nonunion were identified and the nonunion was cleared, decorticated, and bone grafted locally utilising the distal fibula as donor which had been osteotomised and screwed to the talus in the previous tibiotalar fusion procedure. The subtalar joint was prepared by decortication and drilling before being locally grafted. Three 6.5 mm partially threaded cannulated screws were placed to secure the fusion: from the calcaneus to the distal tibia, from the calcaneus to the anterior talus, and posteroanteriorly across the talar nonunion (Figure 4). He was protected non-weight-bearing in a plaster.

He was seen at two weeks postoperatively where wounds were healing satisfactorily and he was immobilised in below knee plaster and reviewed the following week where wounds were fully healed. He was placed into full plaster and seen at six weeks. Radiographs at that time were satisfactory and he was placed into a walker boot and allowed to commence limited weight-bearing.

**Figure 5:**
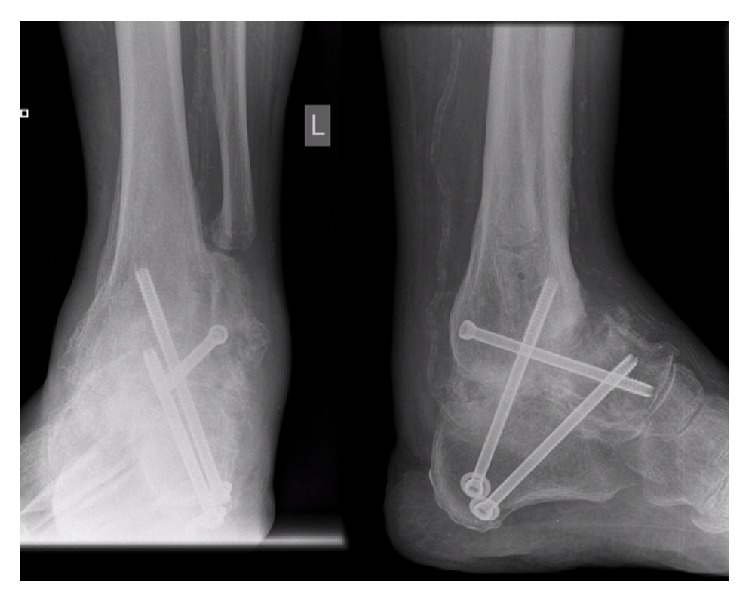
Three-month postsurgery radiographs.

**Figure 6:**
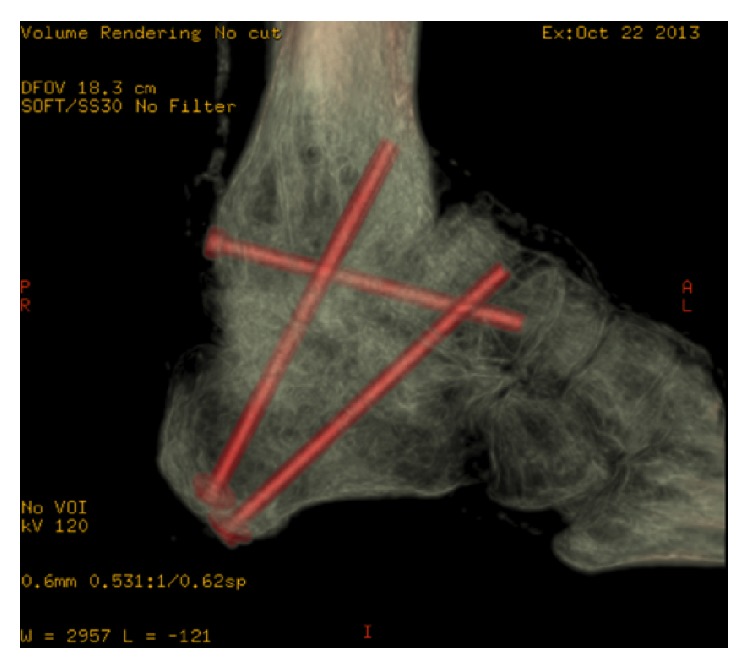
CT demonstrating subtalar fusion and some union across talar neck.

At a three-month postoperative review in October 2013, the patient reported no pain around the ankle and subtalar joint, and the plain radiograph showed progression of union (Figure 5). A CT scan was requested to assess union in more detail (Figure 6) prior to removal of the walker boot.

The patient was reviewed in December 2013, at which time he required no walking aids and was largely pain-free. By the time of final follow-up and discharge from follow-up in February 2014, union was radiologically united and the patient reported being pain-free.

## 2. Discussion

It is important to consider how this fracture occurred in this patient, why it went onto nonunion and pseudarthrosis, and how it was managed.

The usual mechanism of talar neck injuries is that they are the result of high energy trauma, which forces the foot and talus into hyperdorsiflexion [1, 2]. Resultantly, the neck of the talus impinges upon the anterior lip of the tibial plafond and further forced dorsiflexion beyond this point leads to failure at the talar neck through a bending moment about the anterior tibial plafond, which acts as a fulcrum [2, 3]. Clearly, the mechanism in this patient is different, as talar dorsiflexion could not occur. There are several possible mechanisms for this fracture. Firstly, the midfoot and forefoot dorsiflexion upon impact may have caused a bending moment on the tibiotalar fusion. The other alternative is via axial compression transmitted through the calcaneus. The calcaneus did not fracture; therefore, the force may have been transmitted as shear to the talar neck via the posterior subtalar facet, or via the anterior process of the calcaneus. Peterson et al. [4] showed that, in the presence of a fixed ankle joint, a direct impact can give rise to a talar neck fracture. They simulated a direct impact on the sole of a cadaveric foot. Where the ankle was mobile, the fractures involved the bones surrounding the talus (malleolus, calcaneus, etc.) without producing any fractures of the talar neck. Once they eliminated ankle movement, it allowed maximal stress concentration in the talar neck and gave six talar neck fractures in nine consecutive tests. A further study of interest reported the effect of Achilles tension on fracture mechanism in normal ankles. Axial loading may be applied to the leg by an external force, as well as internally, by active muscle tension applied through the Achilles tendon during preimpact bracing. Although calcaneus fractures were the most likely overall fracture, they found that pilon fractures were more likely to occur in the presence of increased Achilles tension [5].

Other authors, in particular, Daniels and Smith [6], had reported that direct blows, extreme direct dorsal force, and supination injuries cause compression against the medial malleolus as alternate mechanisms of sustaining this fracture.

Kwon and Myerson [1] reported on a talar neck fracture in the early postoperative period after tibiotalar fusion. They proposed a low energy mechanism causing the fracture due to osteopenia of immobilisation and furthermore the stress riser effect of recently placed metalwork. In their case, the patient suffered a displaced injury and therefore underwent open reduction and internal fixation. In this case, the period between ankle fusion and the patient sustaining injury was 24 years, certainly adequate time for the patient to load and remodel the bone. Furthermore, his images did not show signs of osteoporosis. This patient also had no metalwork and no stress riser in situ at the time of the injury, with it having been removed 5 years after arthrodesis due to metalwork prominence.

After the likely diagnosis of an undisplaced fracture at the time of injury was missed, this patient was at high risk of developing a nonunion. Firstly, the fracture was not adequately immobilised. Secondly, the blood supply to the fracture would likely be compromised by vasculopathy of diabetes and iatrogenic microvascular damage during surgery. Thirdly, the fused ankle joint would cause extra movement at the fracture site. Fourth, there may have been some mild neuropathy of diabetes (note elevated HbA1C), despite the presence of normal protective sensation. These factors likely account for the development of a painful nonunion/pseudarthrosis.

This case was treated with the use of three screws to compress both subtalar joint and talar neck. The use of a single lateral approach reduced the risk of wound complications in the presence of previous surgeries and scars. The authors were not faced with the technical challenge faced by Kwon and Myerson [1] in their case as there was no immature fusion to preserve.

## 3. Conclusion

A talar neck fracture a long time after tibiotalar fusion has not yet been reported. The likely mechanism is via axial compression of the calcaneus causing shear force across the talar neck. This is an entirely different mechanism to the normal talar neck fracture caused by hyperdorsiflexion of the talus. The fracture has a high risk of nonunion due to the already compromised blood supply to the talus after surgery and due to preferential movement at the fracture site. It is therefore important that this fracture be suspected in any patient presenting with foot and ankle trauma in the presence of a united tibiotalar fusion.
